# Seroprevalence and risk factors of herpes simplex virus-2 among pregnant women attending antenatal care at health facilities in Wolaita zone, Ethiopia

**DOI:** 10.1186/s12985-016-0501-y

**Published:** 2016-03-15

**Authors:** Antehun Alemayehu Anjulo, Tamrat Abebe, Feleke Hailemichael, Adane Mihret

**Affiliations:** School of Medicine, College of Health sciences and Medicine, Wolaita Sodo University, P. O. Box: 138, Wolaita Sodo, Ethiopia; Department of Microbiology, Immunology and Parasitology, College of Health Sciences, Faculty of Medicine, Addis Ababa University, Addis Ababa, Ethiopia; School of Public Health, College of Health sciences and Medicine, Wolaita Sodo University, P. O. Box: 138, Wolaita Sodo, Ethiopia; Armauer Hansen Research Institute, Addis Ababa, Ethiopia

**Keywords:** Herpes simplex virus type-2, Pregnant women, Genital herpes

## Abstract

**Background:**

Herpes simplex virus type-2 is the common cause of genital ulcer disease worldwide. Genital herpes infection is a major concern in pregnancy due to the risk of neonatal transmission.

**Method:**

A Cross-sectional survey was conducted from December 2013 to September 2014 in randomly selected 28 health centers to assess the seroprevalence and risk factors of herpes simplex virus type-2 infection among pregnant women attending antenatal care in Wolaita zone, Southern Ethiopia. After taking written consent socio demographic, behavioral, obstetric history and family planning data along with blood samples were collected from 252 pregnant women using pre-structured questionnaire. Sera were tested using HerpeSelect-2 ELISA IgG. Data entry and analysis was done using Epi info 3.5.4 and SPSS 21.00 respectively. Binary logistic regression was performed to identify the risk factors associated with HSV-2 seropositivity. P-values less than 0.05 were considered statistically significant.

**Results:**

The overall seroprevalence of HSV-2 infection was 32.1 % (81/252) among pregnant women in Wolaita zone. Independent predictors of HSV-2 infection includes daily laborer (AOR 1.293, 95 % CI: 1.033–1.739; *p* = 0.022), having one sexual partners (AOR 0.476, 95 % CI: 0 .250 –0.904; *p* = 0.023), history of STDs (AOR 2.822, 95 % CI: 1.50–5.289; *p* = 0.001) and use of contraceptive (AOR 2.602, 95 % CI: 1.407–4.812; *p* = 0.002).

**Conclusion:**

Overall seroprevalence of HSV-2 infection among pregnant women of Wolaita Zone is high. Awareness creation among high risk groups like women who have history of STD should be strengthened. Strengthening the quality of health service delivery and expansion of health service coverage is mandatory.

## Background

Herpes simplex virus type 2 (HSV-2) is the leading cause of genital ulcer diseases (GUD) worldwide. It is ubiquitous DNA virus belonging to the family of Herpesviridae and it is commonly found in the lumbosacral ganglia in the latent phase [1, 2].

Sexually transmitted diseases (STDs) resulted in stigma and social discrimination in young adult women worldwide [3]. Though incidence of STDs is decreasing, disease caused by HSV-2 infection is overwhelming. Globally 417 million people aged 15–49 years living with HSV-2 infection in 2012, of this 267 million were women. From these infected women 19.2 million were new cases [4]. Global prevalence was higher in women compared to men, especially among young people [5].

In developing countries, it is uncommon to visit health facilities unless having sign and symptom of disease. Prevalence of HSV2 has been observed in higher rate in Sub-Saharan Africa and it occurs more frequently in women (30 to 50 %) than in men (10 to 50 %). More than 80 % of female commercial sex workers were infected with HSV-2 [6, 7].

Neonatal herpes is devastating disease with a 20 % overall mortality; more than 50 % of affected infants have moderate or more severe neurological impairment. Approximately 90 % of all neonatal herpes infections are transmitted during delivery and at least 5 % transmitted in utero. HSV-2 is implicated in more than half (55 %) of neonatal herpes infections [8]. When primary HSV infection occurs during late pregnancy there is not adequate time to produce antibodies before give birth to suppress viral replication. So that the risk of neonatal infection varies from 30 to 50 % if infection is acquired in the late pregnancy but it is around 1 % in the early pregnancy [9].

Recurrent infection is the common character of HSV-2 infection. It favors HIV transmission by disrupting the mucosal barrier and inflammatory changes, which increases recruitment of HIV target cells to the ulcer. Studies indicated that HIV acquisition increased 2 to 4 fold [10, 11]. HSV-2 prevalence is also higher among HIV-infected women; study conducted in Central African Republic showed that HSV-2 antibody prevalence is 91 %. HIV co-infected pregnant women who have clinical genital herpes during pregnancy increases the risk of HIV infection to their infants [12].

HSV-2 infection can be contracted by having sex with infected persons and vertically from mother to foetus/neonates. The first time infection of the mother at third trimester may lead foetus to severe illness and associated with virus transmission from mother to foetus/newborn because of lacking of preexisting antibody [1].

Infections caused by HSV-2 have a higher rate of reactivation. Periodic reactivation from latency is possible and leads to viral shedding from the site of the initial infection. Antiviral therapy does not eradicate latent virus [13, 14].

Individuals infected with HSV-2 are usually asymptomatic but able to transmit the virus to the new born baby. Due to the risk of neonatal transmission assessment of pregnant women sero status is indispensable to treat mother and to make safe new born baby parallel. In addition to that it helps to know the burden of HSV type-2 infection.

The present study aimed to assess the sero prevalence of HSV-2 and associated risk factors among pregnant women attending antenatal care (ANC) in Wolaita zone, Southern Ethiopia, using type specific HSV-2 antibody assay; its structural glycoprotein G (gG-1 in HSV-1 and gG-2 in HSV-2) used to elicit type-specific response [15].

## Methods

### Study area and population

The study was an institution based cross-sectional study conducted from December 2013 to September 2014 in selected health centers in Wolaita Zone, Ethiopia. A total of 252 pregnant women attending antenatal clinics (ANCs) in 28 health centers representing the general population were enrolled in the study. Health centers were stratified based on the place: urban and rural. Selection of health centers were made using simple random sampling method; six from 15 urban health centers and 22 from 55 rural health centers proportionally. Systematically nine study subjects were taken from each selected health center; every seventh client from all pregnant women who seek ANC in selected institution.

### Data and specimen collection

Socio demographic, behavioral, obstetric history and family planning data were collected using pre-structured questionnaire from each participant. Aseptically 5 ml blood was collected using BD Vacutainer blood collection tube.

### Specimen processing

Blood samples were allowed to clot at room temperature prior to centrifugation for 30 min. Serum was separated and transferred to a tightly closing cryo tube vials for storage at −20 °C until further processing. Serum specimens were screened for HSV-2 infection using Herpe Select *2* ELISA IgG test kit (Focus Diagnostics).

Focus Diagnostics’ HerpeSelect *2* ELISA IgG test kit coated with recombinant gG-2 antigen (molecular weight of 80 to 110 kilodaltons) was used for the qualitative detection of human IgG class antibody to HSV-2 [15].

Controls were used to monitor for substantial reagent failure. Vial of human serum with IgG used as positive control which helps to assures reagent functionality and vial of human serum without IgG used as negative control.

### Enzyme linked immune sorbent assay (ELISA)

One hundred milliliter diluted serum samples and controls were incubated in the wells for 1 h at room temperature after covering plates with sealing tape to allow specific antibody present in the samples to react with the antigen. Nonspecific reactants were removed by washing 3× with wash buffer solution and 100 ml peroxidase-conjugated anti-human IgG was added and incubated for 30 min at room temperature. Excess conjugate was removed by washing 3× with wash buffer. 100 ml Enzyme substrate and chromogen was added, and the color was allowed to develop by incubating for 10 min at room temperature. After adding 100 ml Stop Reagent, the resultant color change was quantified by a spectrophotometric reading of optical density (OD) at a wavelength of 450 nm. Sample OD readings were compared with reference cut-off OD readings to determine results.

The cut-off value used to determine a positive test on this kit was greater than1.10. Index value “less than and equal to 1.10 and greater than and equal to 0.9” were considered as equivocal and index values below 0.9 were considered as negative.

To ensure quality each plate run included the cut-off calibrator and all three controls and the result was as the following.

- The mean value for the cut-off calibrator wells was 0.411 OD units.
- The High Positive Control index value was 3.82.
- The Low Positive Control index value was 1.9.
- The Negative Control index value was 0.6.

Index values = Sample optical density/the mean of the Cutoff Calibrator [15].

### Data entry and analysis

Socio demographic and behavioral data entry and analysis was done using Epi info 3.5.4 and SPSS 21.00 version statistical software respectively. Multivariable regression was used to adjust or control the possible confounding factors and to identify risk factors of HSV2 seroprevalence. The cut point for Statistical significance was *P* < 0.05.

### Ethical statement

The study was approved by Ethics and Review Committee of Department of Microbiology, Immunology and Parasitology, School of Medicine Addis Ababa University with protocol number 2/T/2013. Letter of support was obtained from Wolaita Zone Health department. All women who tested positive were communicated their results with the attending health professional and Acyclovir was given. Written consent was obtained from study participants in order to collect different data including biological specimen.

## Results

### Socio demographic characteristics and personal behavior

From all pregnant women who visited ANC during the study period, 252 pregnant women provided the required data and blood samples giving the response rate 100 %. The mean age of the study participants was 24.15 years (SD ± 4.93) and ranged from 15 to 40 years. Majority 170(67.4 %) of the study participants were aged between 20 and 29 years and were Christians 250 (99.2 %). Almost all pregnant women 245(97.2 %) were married. Majority of the study participants were from Wolaita ethnic group, which accounts 221(87.7 %) (Table 1).

**Table 1:** Distribution of ANC attendants by socio demographic and personal behavior variables in Wolaita zone health centers, SNNPR, Ethiopia, 2014

Variables	HSV-2 sero status		Total (%)
	Negative (%)	Positive (%)	
Age			
15–19	23(9.1)	17(6.7)	40(15.9)
20–24	65(25.8)	21(8.3)	86 (34.1)
25–29	54(21.4)	30(11.9)	84(33.3)
> =30	29(11.5)	13(5.2)	42(16.7)
Religion			
Orthodox	51(20.2)	23(9.1)	74(29.4)
Protestant	117(46.4)	58(23)	175(69.4)
Catholic	1(0.4)		1(0.4)
Muslim	2(0.8)		2(0.8)
Marital status			
Married	166(65.9)	79(31.3)	245(97.2)
Single	3(1.2)	2(0.8)	5(2)
Divorce	1(0.4)		1(0.4)
Widowed	1(0.4)		1(0.4)
Ethnicity			
Wolaita	151(59.9)	70(27.8)	221(87.7)
Gurage	6(2.4)	2(0.8)	8(3.2)
Kenbata	1(0.4)	2(0.8)	3(1.2)
Hadya	1(0.4)	0	1(0.4)
Amara	4(1.6)	4(1.6)	8(3.2)
Oromo	3(1.2)	0	3(1.2)
Tigre	5(2)	3(1.2)	8(3.2)
Occupation			
Gov. employee	17(6.7)	10(4)	27(10.7)
House wife	98(38.9)	44(17.5)	142(56.3)
Merchant	21(8.3)	18(7.1)	39(15.5)
Daily laborer	35(13.9)	9(3.6)	44(17.5)
Educational status			
Illiterate	22(8.7)	9(3.6)	31(12.3)
Grade 1–4	27(10.7)	15(6)	42(16.7)
Grade 5–8	45(17.9)	24(9.5)	69(27.4)
Grade 9–12	49(19.4)	15(6)	64(25.4)
Above Grade 12	28(11.1)	18(7.1)	46(18.3)
Alcohol consumption			
No	158(62.7)	73(29)	231(91.7)
Yes	13(5.2)	8(3.2)	21(8.3)
Cigarette smoking			
No	170(67.5)	81(32.1)	251(99.6)
Yes	1(0.4)		1(0.4)
Age at first sex. Intercourse			
15–19	83(32.9)	50(19.8)	133(52.8)
20–24	82(32.5)	25(9.9)	107(42.5)
25–29	6(2.4)	6(2.4)	12(4.8)
Life time No of sex. Partners			
1	94(37.3)	62(24.6)	156(61.9)
> =2	77(30.6)	19(7.5)	96(38.1)
Sex partner now			
No	4(1.6)	3(1.2)	7(2.8)
Yes, regular	165(65.5)	76(30.2)	241(95.6)
Yes, occasional	2(0.8)	2(0.8)	4(1.6)
No of sex. partner last 6 month			
1	167(66.3)	80(31.7)	247(98)
> =2	4(1.6)	1(0.4)	5(2)
History of STD			
Yes	82(32.5)	59(23.4)	141(56)
No	89(35.3)	22(8.7)	111(44)

Almost half of the study participants 133(52.8 %) started first sexual intercourse between age 15 and 19 years. From the study participants, 96(38.1 %) had two or more sexual partners throughout their lifetimes. Five (2 %) of total population had sex with two or more sexual partners in the last 6 months and had new or non-steady sexual partners. One hundred and forty one (56 %) study participants had history of STD. Among the study participants, only 21 (8.3 %) and 1(0.4 %) consume alcohol and smoke cigarette respectively. During the study period, 89(35.3 %) study participants got Provider Initiated Testing and Counseling service (PITC) and 1(1.1 %) was positive for HIV (Table 1).

### Obstetric history and family planning

In terms of obstetric history, majority of the study participants 187(74.2 %) were at age greater than or equal to 18 years at first pregnancy and the rest were less than 18 years. From all study participants, majority 145(57.5 %) had more than three children. The median gestation week of the participants was 28 weeks and ranged from 4 to 40 weeks (SD ± 7.775), majority of study subjects, 141(56 %) had 20–28 weeks gestational period.

Among all study participants, only 30(11.9 %) had history of abortion. With regard to contraceptive, 141 (56 %) had been using contraceptive before being pregnant. In the present study, HSV-2 was high among pregnant women whose age were greater than or equal to 18 years at first pregnancy, mother having “no birth-2” children, gestational weeks between 20 and 28 weeks, women who had no history of abortion and used contraceptive which accounts 54(66.6), 38(46.9),44(54.3), 71(87.6) and 59(72.8 %) respectively (Table 2).

**Table 2:** Distribution of ANC attendants by Obstetric history and family planning in Wolaita Zone health centers, SNNPR, Ethiopia, 2014

Variables	HSV-2 sero status		Total (%)
	Negative (%)	Positive (%)	
Age at 1^st^ pregnancy			
< 18	38(15.1)	27(10.7)	65(25.8)
> =18	133(52.8)	54(21.4)	187(74.2)
No of child			
0–2	69(27.4)	38(15.1)	107(42.5)
3–5	65(25.8)	23(9.1)	88(34.9)
> 5	37(14.7)	20(7.9)	57(22.6)
Gestational wks			
< 20	9(3.6)	6(2.4)	15(6)
20–28	97(38.5)	44(17.5)	141(56)
> 28	65(25.8)	31(12.3)	96(38.1)
Abortion			
Yes	20(7.9)	10(4)	30(11.9)
No	151(59.9)	71(28.2)	222(88.1)
Used contraceptive			
Yes	82(32.5)	59(23.4)	141(56)
No	89(35.3)	22(8.7)	111(44)

### HSV-2 seroprevalence and risk factors

The overall seroprevalence of HSV-2 infection in Wolaita Zone, Ethiopia was 32.1 % (81/252) among pregnant women. Independent predictors of HSV-2 infection includes daily laborer (AOR 1.293, 95 % CI: 1.033–1.739; *p* = 0.022), having one sexual partners (AOR 0.476, 95 % CI: 0 .250 –0.904; *p* = 0.023), history of STDs (AOR 2.822, 95 % CI: 1.50–5.289; *p* = 0.001) and use of contraceptive (AOR 2.602, 95 % CI: 1.407–4.812; *p* = 0.002), these variables were used to form multivariable logistic regression analysis (Table 3). The cut point for Statistical significance was *P* < 0.05.

**Table 3:** Bi variable models of factors associated with HSV-2 prevalence among pregnant women attending ANC in Wolaita zone health centers, SNNPR, Ethiopia, 2014

Variables	HSV-2 sero status		COR	95 % C.I. for COR		*P*- value
	Negative (%)	Positive (%)		Lower	Upper	
Age						
> =30 year	29(11.5)	13(5.2)	1			
15–19 years	23(9.1)	17(6.7)	1.649	0.666	4.080	0.279
20–24 years	65(25.8)	21(8.3)	0.721	0.318	1.634	0.433
25–29 years	54(21.4)	30(11.9)	1.239	0.561	2.736	0.595
Educational level						
Above grade 12	28(11.1)	18(7.1)	1			
Illiterate	22(8.7)	9(3.6)	0.636	0.240	1.688	0.364
Grade 1–4	27(10.7)	15(6)	0.864	0.364	2.053	0.741
Grade 5–8	45(17.9)	24(9.5)	0.830	0.383	1.795	0.635
Grade 9–12	49(19.4)	15(6)	0.476	0.208	1.090	0.079
Occupation						
Merchant	21(8.3)	18(7.1)	1			
Gov.employee	17(6.7)	10(4)	0.686	0.252	1.872	0.462
Housewife	98(38.9)	44(17.5)	0.524	0.254	1.079	0.080
^a^Daily labours	35(13.9)	9(3.6)	2.410	1.114	3.788	0.015
1^st^ sexual intercourse age						
^a^25-29 years	6(2.4)	6(2.4)	1			0.028
15–19 years	83(32.9)	50(19.8)	0.602	0.184	1.970	0.402
20–24 years	82(32.5)	25(9.9)	0.305	0.090	1.030	0.056
Life time no of sex. partner						
1	94(37.3)	62(24.6)	0.374	0.206	0.679	0.001
^a^ > =2	77(30.6)	19(7.5)	1			
STI symptoms						
no	89(35.3)	22(8.7)	1			
^a^ yes	82(32.5)	59(23.4)	2.911	1.639	5.169	0.000
Gestation wks						
> 28 weeks	65(25.8)	31(12.3)	1			
< 20 week	9(3.6)	6(2.4)	1.398	0.457	4.276	0.557
20–28weeks	97(38.5)	44(17.5)	0.951	0.545	1.660	0.860
Age at 1^st^ pregnancy						
> =18 years	133(52.8)	54(21.4)	1			
< 18 years	38(15.1)	27(10.7)	1.750	0.974	3.144	0.061
Contraceptive						
No	89(35.3)	22(8.7)	1			
^a^ Yes	82(32.5)	59(23.4)	2.911	1.639	5.169	0.000
No of child						
> 5	37(14.7)	20(7.9)	1			
0-2	69(27.4)	38(15.1)	1.019	0.520	1.997	0.957
3–5	65(25.8)	23(9.1)	0.655	0.318	1.348	0.250

^a^Statistical significant association

Multivariable logistic regression analysis revealed that daily laborers were 1.29 times likely to be positive for HSV-2 than merchants (*P* = 0.022, AOR 0.293, 95 % CI: 0.103–0.839), and having one sexual partners were 52.4 % less likely to be positive than having two or more sexual partner (*p* = 0.023, AOR0.476, 95 % CI: 0 .250 –0.904).

The seroprevalence of HSV-2 was 2.8 times higher among Women who had a history of STD (*p* = 0.001, AOR 2.822, 95 % CI: 1.50–5.289) and in contraceptive users it was 2.6 times high (*p* = 0.002, AOR 2.602, 95 % CI: 1.407–4.812). However, the association of age at first sex disappeared in the multivariate analysis (Table 4).

**Table 4:** Multivariable models of factors associated with HSV-2 prevalence among pregnant women attending ANC in Wolaita zone health centers, SNNPR, Ethiopia, 2014

Variables	HSV-2 sero status		AOR	95 % C.I. for AOR		*P*-value
	Negative (%)	Positive (%)		Lower	Upper	
Occupation						
Merchant	21(8.3)	18(7.1)	1			
Gov. employee	17(6.7)	10(4)	0.686	0.209	2.254	0.535
Housewife	98(38.9)	44(17.5)	0.593	0.262	1.341	0.210
^a^Daily labours	35(13.9)	9(3.6)	1.293	1.033	1.739	0.022
1^st^ sexual intercourse age						
25–29 years	6(2.4)	6(2.4)	1			
15–19 years	83(32.9)	50(19.8)	0.704	0.170	2.917	0.628
20–24 years	82(32.5)	25(9.9)	0.339	0.080	1.433	0.141
Life time no of sex. partner						
^a^1	94(37.3)	62(24.6)	0.476	0.250	0.904	0.023
STI symptoms						
^a^yes	82(32.5)	59(23.4)	2.822	1.50	5.289	0.001
Contraceptive						
^a^yes	82(32.5)	59(23.4)	2.602	1.407	4.812	0.002

^a^Statistical significant association

## Discussion

In this study we found that 32.1 % (81/252) overall sero prevalence of HSV-2 among pregnant women attending antenatal care; which was higher as compared to studies done in a similar study population in different countries, in USA 22 %, 7.6 % in Italy,14.5 % in Australia, 8.7 % in Northeast India and 20.7 % in a rural area of Tanzania [16–20].

The higher prevalence in our study might be due to various factors. First, the prevalence of HSV-2 might be high among the general population especially in men. Financial constraints might be the second factor as it was difficult to cover screening cost in our country and simultaneously creating awareness. For example, there were no laboratory materials in the health centers to screen HSV-2 in our country. Educational status might be the other factor where majority of women in reference countries had better educational status than in Ethiopia. Place of residence might have also played an important role. The present study was done in southern part of Ethiopia where we included many health centers from rural areas. The areas had poor health facility, lack of transportation to health facility and lack of health information might play important role to increase the prevalence.

But the prevalence was lower from a study done in Zimbabwe amongst women of childbearing age at high risk group, which was 49.1 % [21]. This difference could be due to the difference in the study group where we were not focused only on high risk group. However, the study done in Zimbabwe encompassed high risk group women.

Daily laborers had higher HSV-2 prevalence, and they were 1.293 times more likely to be positive than Merchant. This is in line with a study done in different countries [22, 23]. This might be due to the fact that women were working in high risk settings, like selling coffee, tea and bread on the street, and waitress in bars and this working environment might increase the risk to exposure to multiple sexual partners.

Significant association was observed in the crude analysis among participants who started first sexual intercourse between age 25–29 years (*p* = 0.028). However, this association was lost in the multivariate analysis after controlling occupation, life time number of sexual partner, STI symptoms and contraceptive use.

Significant increase for the risk of acquiring HSV-2 infection was observed for women who had multiple sexual partners, compared with women who had single sexual partner (*P* = 0.001). It was similar with many studies conducted in Zimbabwe, USA, North East India and Tanzania [2, 19–21]. This might be due to increased frequency of exposure to risky sexual intercourse.

When we see history of STD, strong association was observed among participants who had STI symptoms, compared with women who mentioned never encountered STI symptoms (*p* = 0.00). This is similar with a study conducted in Mexico which showed significant associations in the crude analysis among participants who had gonorrhea (OR = 1.7, 95 % CI: 1.1–3.0) and those individuals who tested positive for exposure to T. *pallidum* (OR = 2.8, 95 % CI: 1.7–5.0) [23]. This might be due to disrupted mucosal barrier and inflammatory change.

When we see the association between HSV-2 and hormonal contraceptive users, women who ever used hormonal contraceptive in their life time had strong association with the risk of acquiring HSV-2, compared with never used contraceptive before (*p* = 0.000).It is the same with a study in Mozambique, Beira where hormonal contraceptive users had higher risk in acquiring HSV-2 AOR 1.92 (CI: 1.16–3.19, *P* = 0.012) [24]. This might be because of either they have had sex frequently with infected individuals or immune suppression. Cell-mediated immunity, which is important for control of viral infections, is depressed by both estrogen and During pregnancy, serum estradiol and progesterone concentrations increase steadily, ultimately reaching levels that are 10 to 100 fold higher than those occurring during normal menstrual cycling [25].

The present study confirmed that HSV-2 prevalence among pregnant women attending antenatal care in Wolaita zone health center was high and this might increase risk of neonatal transmission. A study in Italian pregnant women showed that 3 % women acquired HSV infection during pregnancy. In USA 2 % pregnant women and 2.6 % in Norway acquired the infection close to term and placing their newborn at risk for herpes infection during delivery. The risk of infection varies from 30 to 50 % during late pregnancy however it is 1 % in early pregnancy [9, 26, 27].

## Conclusion and recommendation

In the present study the Sero-prevalence of HSV-2 among pregnant women was high. Being daily laborer, having two or more sexual partners, history of STD and using hormonal contraceptive were independent predictors for HSV-2 sero-prevalence. The risk of neonatal transmission could be high because of high sero-prevalence among pregnant women. Women who have history of STD should be tested and treated early. Health campaigns among women should promote condom use; bring behavioral change and introduction of STI screening. Strengthen the quality of health service delivery and expand health service coverage. To access STD clinics minister of health needs to work without any reservation.
